# Navigating behavioral energy sufficiency. Results from a survey in Swiss cities on potential behavior change

**DOI:** 10.1371/journal.pone.0185963

**Published:** 2017-10-09

**Authors:** Roman Seidl, Corinne Moser, Yann Blumer

**Affiliations:** 1 ETH Zurich, D-USYS Transdisciplinarity Lab, Zurich, Switzerland; 2 ZHAW School of Engineering, Institute of Sustainable Development, Winterthur, Switzerland; 3 ZHAW School of Management and Law, Center for Innovation and Entrepreneurship, Winterthur, Switzerland; Universita degli Studi della Tuscia, ITALY

## Abstract

Many countries have some kind of energy-system transformation either planned or ongoing for various reasons, such as to curb carbon emissions or to compensate for the phasing out of nuclear energy. One important component of these transformations is the overall reduction in energy demand. It is generally acknowledged that the domestic sector represents a large share of total energy consumption in many countries. Increased energy efficiency is one factor that reduces energy demand, but behavioral approaches (known as “sufficiency”) and their respective interventions also play important roles. In this paper, we address citizens’ heterogeneity regarding both their current behaviors and their willingness to realize their sufficiency potentials—that is, to reduce their energy consumption through behavioral change. We collaborated with three Swiss cities for this study. A survey conducted in the three cities yielded thematic sets of energy-consumption behavior that various groups of participants rated differently. Using this data, we identified four groups of participants with different patterns of both current behaviors and sufficiency potentials. The paper discusses intervention types and addresses citizens’ heterogeneity and behaviors from a city-based perspective.

## 1 Introduction

Nations around the world plan to reduce their fossil fuel—based energy usage and related carbon emissions to combat climate change [1, 2]. With the Paris Agreement on Climate Change, the 197 participating countries officially acknowledged the need for clear action [3]. European countries in particular have recently defined transformation targets for their existing energy regimes [4–6]. Technological measures alone will likely be insufficient to reach these ambitious reduction goals in energy consumption and emissions; societal changes (via residents’ behavioral changes) are also necessary [7–9]. This situation especially affects OECD (Organization for Economic Cooperation and Development) countries with high living standards in which households account for a substantial share of national energy use. Behavioral changes at the individual level thus can reduce energy demand. Through such behavioral changes and increased efficiency, individuals can substantially reduce their energy consumption and carbon emissions [10].

One such country is Switzerland, where households were responsible for 26.5 percent of final primary-energy usage in 2014 [11], which was higher than the EU-28 average of 24.8% in the same year (source: Figure 3 in [12]). Switzerland’s new energy strategy has ambitious goals to promote renewable energy sources as substitutes for nuclear energy and fossil fuels as well as reductions in per-capita consumption [13]. Switzerland expects an 18% decrease in average per-capita electricity consumption by 2050 compared to the base year 2000. The nation’s per-capita reduction targets for energy consumption for mobility and heating are even more ambitious [13], as both areas together account for the majority of total energy consumption; for instance, private transport accounts for more than one-third of final energy consumption in Switzerland [14]. Improvements that will enhance energy efficiency (of appliances, cars, and buildings, for example) are components of achieving a more sustainable energy system. Besides the technological aspect of an energy-system transformation, however, citizen consumers’ behavioral changes (usually denoted as “sufficiency”) constitute important components as well. Indeed, research on efficiency measures is still more prominent than that on behavior-related sufficiency approaches. For instance, using the search words “energy,” “sufficiency,” and “behavior” in Google Scholar yields fewer results (approximately 105,000); instead, the engine even suggests searching for “energy,” “efficiency,” “behavior,” which yields 3,590,000 hits (Date of search: July 11, 2016). This is especially true because studies have identified various rebound effects of energy-efficiency approaches [15, 16]. In accordance with Breukers et al.’s definition [17], we consider “energy sufficiency” as a change in everyday life routines and cultural conventions that leads to lower energy consumption. Examples of energy-sufficient behavior include switching to a vegetarian diet, wearing a sweater and warm socks in the winter (instead of turning up the heat in an apartment), using public transport instead of a private car for commuting, and sharing common rooms.

One important approach to realizing such sufficiency potentials involves interventions that attempt to influence individual behavior [18]. Interventions can be an effective tool to address behavioral changes to being more sustainable [19, 20], although evidence from many intervention programs has shown the difficulty of recruitment, especially among residents who are less environmentally conscious [21, 22].

Various studies have examined interventions aimed at energy-efficient and energy-sufficient behavior [20, 23]. For example, Abrahamse et al. [20] distinguish between antecedent and consequent interventions that promote energy conservation. For the former interventions, commitment and goal setting are successful strategies; for the latter, certain incentives or rewards are effective despite the risk that the participants may revert to prior behaviors with the termination of the intervention. For general energy-saving behavior, Poortinga et al. [24] have found that people generally prefer technical efficiency measures over behavioral changes; at the same time, people favor energy-saving measures at home over measures that affect their mobility. A recent Swiss study using conjoint analysis identified several varieties of the willingness to adopt sufficiency measures in different domains [25]. For instance, while most participants rejected a strict vegetarian diet, substantial sufficiency potentials seemed to exist in the domains of household room temperature, personal leisure travel behavior, and size of per-capita living spaces. That study analyzed the sample as a whole and did not consider subgroups.

Many of the interventions discussed above are implemented by local authorities rather than by national governments. A city government, in particular, is closer to its inhabitants and to citizen consumers than would be the case with a national government. Cities may serve as important actors because of their assigned role as agents of change in Switzerland’s new energy strategy [13]. Cities may contribute to this strategy in two ways. They may serve as role models by promoting energy efficiency and sufficiency, such as by participating in the European Energy Award (EEA) program (by having energy-efficient public buildings, for example). They may directly motivate residents as well, for instance through energy-saving campaigns. Cities have attempted to change citizens’ energy-related behavior for many years, such as by reducing the impact of mobility behavior (particularly the use of private transport) [26, 27]. An increasing number of European cities strive for energy labels such as the EEA Gold [28]. Cities may engage in such interventions to reduce energy consumption in order to avoid infrastructural costs or to comply with political mandates. Many Swiss cities’ commitments to promoting less energy-consuming lifestyles have actually been legitimized by popular votes. Consequently, many of the larger Swiss cities have adopted the goals of the so-called 2,000W society [29] one of which is to curb citizens’ per-capita primary-energy consumption to 2,000 watts [30].

For cities to design successful behavioral-change interventions, however, it is essential to tailor the interventions to different target groups [31–33] and to consider variations in their willingness to change their behavior. Sütterlin et al. [22] identified six energy-consumer segments in their study on energy conservation, as follows: (1) idealistic energy saver (15.6%); (2) selfless, inconsequential energy saver (26.4%); (3) thrifty energy saver (14%); (4) materialistic energy consumer (25.1%); (5) convenience-oriented, indifferent energy consumer (5.3%); and (6) problem-aware, wellbeing-oriented energy consumer (13.6%). Another study [34] found different lifestyle groups in terms of their environmental actions; the authors identified four clusters, including committed and mainstream environmentalists and occasional and non-environmentalists.

While these studies refer to the behavioral status quo, another line of research addresses the potential genesis of sustainable behavior over time. Bamberg [35] proposes a more differentiated stage model vis-à-vis diverse target groups. Referring to Gollwitzer’s [36] model of action phases, Bamberg suggests that individuals can be assumed to be at different stages of their decision process in terms of environment-related behavioral change. He distinguishes the four stages as pre-decision, pre-action, action, and post-action and highlights three transition points for behavioral change: goal, behavioral, and implementation intentions. For instance, the “goal” intention point is located at the pre-decision stage, when factors such as norms and perceived individual responsibility are relevant. Ohnmacht et al. [37] recently provided recommendations for operationalizing meat consumption (as one example) for each stage of the authors’ framework.

A city’s campaign to raise awareness and the perception of responsibility thus would only consider individuals at this stage of behavioral change, but it is inappropriate to reach those people who already acknowledge their own responsibility but feel hampered at the implementation-intention stage. Gardner and Stern’s [23] short list of energy-saving activities or measures to inform citizens about the possibilities and their respective energy-saving potentials may be helpful for those who are aware of the energy problem or who already intend to change their behaviors. For people at the earlier stages of behavioral change, however, this list only provides households with factual and practical knowledge to help residents adapt their behavior accordingly, but not by raising awareness or highlighting social norms.

For our work, we stress tailoring to target groups as an important aspect of successful interventions and aim for a combination of both temporal aspects (stages) and citizens’ heterogeneity. Hence, we may assume that different groups of people are at various stages of behavioral change and that some may respond better to either antecedent or consequent interventions. Because cities must consider both aspects when planning interventions, we addressed both issues in an online questionnaire and related these issues to cities’ interventions to foster sufficiency behavior [30]. In this paper, we thus seek answers to the following questions:

1. From an individual perspective, where does the potential exist for behavioral changes via cities’ interventions? Could any thematic sets of behaviors be addressed by an intervention?
2. Can different groups of individuals be classified by their current behaviors/activities, conceptualized as independent variables? Are there any links between these behaviors and future behavioral-change potentials, conceptualized as dependent variables?

## 2 Method

### 2.1 Sample description

This study was undertaken within a collaborative project among the research team and three Swiss cities that had already received gold status according to the above-mentioned EEA label. Although the three cities had experience in energy reductions, they assumed their citizens had further potential.

We conducted an online survey in the three Swiss cities of Baden (*N* = 83), Winterthur (*N* = 422), and Zug (*N* = 201), resulting in a total of 706 usable cases for analysis. The survey can be accessed as supporting information both in German (original, S1 File) and English (translation, S2 File). All three cities are in the German-speaking part of Switzerland. The samples were recruited by an online panel (Respondi, see [38]). Because the panel members were paid by Respondi, their consent to the present study may be assumed. The data was analyzed anonymously. In addition, this kind of non-invasive research is considered to be category A at the first author’s institution and thus does not require approval by the Ethics Commission [39].

No significant differences among the city samples could be found with the independent variables (see Section 2.1), and only marginal variations among the three cities were observed among the dependent variables (see Section 2.3). One marginally significant difference was found in car sharing (using multiple comparison via the Bonferroni technique, 99% level): the inhabitants of Zug seemed to have a particular affinity for cars. Thus, the results for this variable differed because the Winterthur participants would more readily share cars. The merged sample’s mean age was 51 years, with a minimum of 18 and a maximum of 99 (the mean age in Switzerland is 42; see [40]). Because 48% of the participants were female, the gender sample did not perfectly match the Swiss population, around half of whom (50.5%) are women [41]. The average living space was 51 m^2^ per person (standard deviation [SD] = 22.76). Approximately two-thirds (66.5%) of the sample owned a car, and 72.4% reported having a public transport subscription (PTS); 60% of the sample indicated having one or more memberships in various associations (sports and the like).

### 2.2 Independent variables: Current behaviors/activities

For the independent variables, we collected data on the importance of the participants’ current behaviors/activities, represented by 14 energy-related behaviors, habits, or situations in the form of questions, each starting with “How important is it for you to…?” The questions were rated on a 7-point Likert scale, ranging from 1 (high sufficiency) to 7 (low sufficiency; see Table 1).

**Table 1:** Item text and statistics of the independent variables. Sorted according to decreasing sufficiency (lower values denote higher sufficiency, rated on a scale from 1 to 7) and increasing mean values.

How important is it for you to…	Mean	SD
take a bath more than once a week?	2.1	1.73
consume goods that are not produced locally?	2.6	1.45
live far away from work?	2.8	1.60
take a long shower?	3.1	1.63
sleep with an open window during the winter?	3.3	2.08
own a car?	3.3	2.20
have your electronic devices always on standby?	3.4	1.85
go on vacation to remote countries?	3.7	1.93
consume meat?	3.7	1.69
change your clothes daily (e.g., a sweater)?	3.8	1.85
be comfortably warm without a sweater during the winter?	3.9	1.77
perform your daily tasks (work, study, etc.) at the workplace and not at home?	3.9	1.77
have a large living area at your disposal?	4.4	1.45
own tools and household items?	4.8	1.61

As part of the collaboration with the cities, the following demographic variables of interest for each city administration were included because of their potential relevance for the social stratification or grouping of the participants: car ownership in each household (yes/no), PTS (yes/no), home ownership, and membership in associations. This last variable was included because of the cities’ interest in whether collaboration with associations, as examples of formal social groups, would make sense in terms of interventions.

### 2.3 Dependent variables: Behavioral-change potentials

The items we used to measure behavioral-change potential had a specific format. We encouraged the participants to compare their attitudes with those of two fictitious characters, “Tony” and “Mira.” The characters either espoused more or less energy-sufficient activities, or both opted for a certain activity. The participants were asked to express how they related to the characters’ respective preferences or choices. This procedure drew on the successful application of the “Portraits Value Questionnaire” [42, 43], where respondents compared individuals described in the variables with themselves and then rated the perceived similarities. In a comparable manner, we asked the respondents whether they identified more with Tony or Mira and whether or not they could personally relate to the lifestyles the characters espoused (items developed in [44]).

In the first item set, the Tony and Mira characters represented sufficient and nonsufficient activities. A sample item stated, “Mira thinks the apartment should be heated only to a degree where she feels comfortable wearing a pullover. Tony doesn’t agree with Mira at all; he wants to have it warm so that wearing a T-shirt would be sufficient.” The respondents were asked to reveal, on a 7-point scale, with whom they agreed more, Mira or Tony. One of the options was always less energy-sufficient (7 on the rating scale) than the other. The items randomly alternated genders, so there was no stable pattern of, for example, Mira = female = sufficient.

A second item set presented Tony and Mira performing joint activities; for instance, “Tony and Mira are looking for a new apartment. They have accepted an offer where the apartment is smaller than their current one, but they can use guest rooms and lounges in the building if needed.” The participants were then asked how attractive this scenario was for them. They rated the variables from 1 = very attractive (high sufficiency) to 7 = not at all attractive (low sufficiency).

Table 2 presents the mean values and standard deviations for all variables (the one item on commuting appeared unusable for analysis; it failed to yield any relevant information or correlate with any other item and was thus omitted from further analysis and the tables (but see the full survey in the supporting information, S1 and S2 Files). Commuting most likely differs too much from the other items and depends on participants’ personal situations). For instance, the respondents acknowledged switching off the lights and purchasing regional products as fairly frequent activities, whereas they rated sharing rooms in the context of reduced living space and giving up vacations to distant places less highly, thus indicating a less agreed-on behavioral change.

**Table 2:** These variables indicate potential future activities and serve as dependent variables, presented as scenarios with the fictitious characters Tony and Mira in two different item sets (see main text).

Variable (Tony and Mira)	Mean	SD	Item set
Airing rooms (intermittent ventilation)	1.6	2.00	2
Switching off lights	2.1	2.02	1
Purchasing regional products	2.1	1.55	2
Sharing tool kits	2.5	1.83	2
Car sharing	2.7	1.98	2
Reducing room temperature	2.8	2.06	1
Washing	3.0	2.31	1
Showering	3.0	1.27	1
Consuming meat	3.2	2.13	1
Working at home	3.6	1.39	1
Having a large living space	3.7	1.67	2
Going on vacation to faraway places	4.0	1.96	1

The variables are sorted according to increasing mean values. The lower the sufficiency potential, the higher the potential for behavioral change. Lower values denote higher sufficiency (on a scale of 1 to 7).

### 2.4 Statistical analyses

We used factor analysis (alpha factoring with Varimax rotation) to analyze the structure of the dependent variables. Using this approach, we identified four thematically distinctive factors. We also conducted different statistical measures and tests. For the independent variables illustrated in Section 2.1, we performed a cluster analysis [45, 46], using the Ward method with squared Euclidean distance and a standardization of the items to a range from 0 to 1 [47]. The Ward method attempts to calculate equally sized clusters. We used the exploratory technique of cluster analysis to investigate whether groups of respondents could be characterized by their distinctive ratings of the independent variables. Three different cluster solutions were evaluated (three-, four-, and five-cluster solutions). Finally, we opted for the four-cluster solution, because it revealed the clearest results while maintaining fairly equal numbers of participants in each cluster.

We then characterized the resulting clusters according to the independent variables and sociodemographic data (Section 3.2); we also compared the clusters in terms of the dependent variables related to the sufficiency potential (Section 2.3). We used analysis of variance (ANOVA) with Bonferroni multiple comparison (99%) to test for the statistical significance of any differences among the clusters (Section 3.2.3).

## 3 Results

### 3.1 Descriptive results

Table 3 shows the results of the factor analysis, summarizing the dependent variables in four factors. The first and most important factor comprises sharing of cars and tools, as well as the size (in square meters) of the living space. Meat consumption also loaded on this factor, although the value was comparatively low; thus, it may be related to (but not essential for) defining the factor. Factor 1 could be termed “possession of goods versus sharing” and could be important to ideas for a sharing economy. Factor 2 describes behaviors at home that are related to energy consumption; these are essentially established daily behaviors such as using ventilation (i.e., opening the windows for a quick ventilation as opposed to tilting windows for a longer time) or switching off the lights when leaving a room. Factor 3 relates to washing and hygienic standards. Note that this item does not significantly load on Factor 2 and thus should be regarded as distinct. Factor 4 involves working at home, which (similarly to Factor 3) may depend on specific contexts. The elements of such contexts include washing-machine ownership, job type, an employer’s openness to working at home, hygienic aspects, and lifestyle expectations. To illustrate this factor, consider the wording of the question on washing: “Tony wears the same sweater for several days. Mira prefers to wear a freshly washed sweater every day.” The variable “going on vacation to faraway places” could not be assigned to any of the four factors, since it loaded very low on each (between –0.12 and 0.32).

**Table 3:** Results (rotated factor matrix) of a factor analysis. Factor loadings of the variable “going on vacation to faraway places” were all below 0.35. All values below this threshold were omitted in this table (see, [48]).

Variable	Factor			
	1	2	3	4
Car sharing	0.68			
Sharing tool kits	0.59			
Having a large living space	0.49			
Consuming meat	0.36			
Going on vacation to faraway places	-	-	-	-
Reducing room temperature		0.49		
Showering		0.48		
Switching off lights		0.46		
Purchasing regional products		0.39		
Airing rooms (intermittent ventilation)		0.37		
Washing			0.65	
Working at home				0.45
*Eigenvalues*	*3*.*11*	*1*.*18*	*1*.*07*	*1*.*03*
*% of variance*	*25*.*91*	*9*.*86*	*8*.*89*	*8*.*62*

Extraction method: alpha factoring. Rotation method: varimax with Kaiser normalization. Rotation converged in five iterations.

### 3.2 Cluster analysis of independent variables

#### 3.2.1 Independent variables

To identify groups of people who displayed similar behavioral patterns, we used the independent variables (Section 2.1) for a cluster analysis and tested for differences among the clusters (regarding both independent and dependent variables). Table 4 shows the differences among the clusters for the independent variables. With the exception of two items (both related to the distance to a respondent’s workplace), an overall ANOVA revealed significantly different clusters (*r* < 0.001). Table 4 indicates specific significant differences (see letters a–d, corresponding to Clusters 1–4, respectively).

**Table 4:** Results of the cluster analysis of independent variables.

How important is it for you to…	Cluster 1: Homeowners with car affinity (*N* = 156)^a^		Cluster 2: High standard of living but conscious consumption (*N* = 89)^b^		Cluster 3: Sufficient consumption (*N* = 149)^c^		Cluster 4: Middle group with potential (*N* = 208)^d^	
	Mean	SD	Mean	SD	Mean	SD	Mean	SD
have a large living area at your disposal?	5.2^b,c,d^	1.22	4.4^a,c^	1.34	3.6^a,b,d^	1.41	4.3^a,c^	1.36
be comfortably warm without a sweater during the winter?	5.3^b,c,d^	1.18	3.8^a,c^	1.79	2.8^a,b,d^	1.55	3.7^a,c^	1.65
sleep with an open window during the winter?	4.3^b,d^	1.98	2.1^a,c^	1.54	4.1^b,d^	2.15	2.4^a,c^	1.70
perform your daily tasks (work, study, etc.) at the workplace and not at home? ^ns^	4.2	1.78	3.8	1.89	3.8	1.71	3.9	1.74
go on vacation to faraway countries?	4.5^b,c^	1.82	3.5^a,c,d^	1.78	2.3^a,b,d^	1.26	4.3^b,c^	1.86
own tools and household aids?	5.4^c,d^	1.40	5.4^c,d^	1.52	4.6^a,b^	1.71	4.4^a,b^	1.57
own a car?	5.3^c,d^	1.59	5.4^c,d^	1.35	2.1^a,b^	1.60	1.9^a^	1.27
take a bath more than once a week?	3.2^b,c,d^	2.10	1.6^a^	1.35	1.3^a,d^	0.66	1.9^a,c^	1.66
consume meat?	4.4^c,d^	1.70	4.0^c^	1.77	2.8^a,b,d^	1.33	3.7^a,c^	1.60
wear freshly washed clothes (e.g. a sweater), aside from underwear?	5.1^b,c,d^	1.48	3.1^a,d^	1.72	2.6^a,d^	1.34	3.9^a,b,c^	1.78
take long showers?	3.9^b,c,d^	1.58	2.6^a,d^	1.51	2.4^a,d^	1.32	3.2^a,b,c^	1.61
live far away from work? ^ns^	2.9	1.62	2.6	1.56	2.8	1.55	2.9	1.63
put devices on standby mode?	3.2	1.82	3.7^c^	1.89	2.4^b,d^	1.41	4.1^a,c^	1.81
consume goods that are not produced locally?	2.8^c^	1.41	2.7	1.50	2.2^a,d^	1.35	2.7^c^	1.46

All cluster differences are significant at the 99% interval (ns = not significant). For multiple comparisons, we indicate the significance by superscript letters: Cluster 1 = a, Cluster 2 = b, Cluster 3 = c, Cluster 4 = d.

According to these results, we denoted Cluster 1 as homeowners with car affinity, a high standard of living, and a relatively high need for a spacious living area, freshly washed clothes, and so on. Cluster 2 was somewhat similar in terms of ownership of tools and cars but had lower values for other variables, such as bathing and showering; having their own living areas and a warm apartment during the winter; and using ventilation and switching off lights. Cluster 3 showed comparatively very low values in every respect and thus had fairly sufficient behavior/activity levels. Cluster 4 was somewhat in between the other clusters and resembled the items’ mean values (i.e., the whole sample’s average).

#### 3.2.2 Demographic description of clusters

The cluster analysis yielded four distinctive clusters with somewhat equal sizes. To better interpret the results, we present a brief demographic description of the clusters in Table 5. The most obvious difference in the respondents’ gender occurred between Clusters 1 and 2 (~60% males for both) and Clusters 3 and 4 (44–46% males for both). Cluster 4 differed significantly in the mean age (46 years) from Clusters 2 (50 years) and 3 (55 years) but not from Cluster 1 (53 years); an ANOVA test yielded a significant result (F_(3, 521)_ = 27.2, *p* < 0.01).

**Table 5:** Demographic description of the four clusters.

Demographic variable		Cluster 1: Homeowners with car affinity (*N* = 142)^a^	Cluster 2: High standard of living but conscious consumption (*N* = 83)^b^	Cluster 3: Sufficient consumption (*N* = 136)^c^	Cluster 4: Middle group with potential (*N* = 193)^d^
Car ownership in household (yes/no)		94%	93%	51%	46%
Public transport subscription (yes/no)		62%	55%	79%	82%
Home ownership		46%	54%	46%	21%
Membership in one or more associations		54%	65%	63%	61%
Number of persons per household	1	20%	17%	21%	30%
	2	49%	45%	43%	43%
	3 or more	30%	38%	35%	27%
Gender (female)		40%	39%	54%	56%
Mean age (years)		53	50	55	46
Children in household (yes/no)		20%	26%	23%	14%
Monthly household income in % of total within income category	≤ 8,000 CHF	32%	36%	43%	40%
	8,001–14,000 CHF	20%	33%	35%	32%
	> 14,000 CHF	17%	13%	6%	11%

^a,b,c,d^: Note that income characterization is limited because 113 participants did not reveal their incomes (Cluster 1: 43; Cluster 2: 15; Cluster 3: 22; Cluster 4: 33). All respondents also had to indicate if their households owned a car and if they had PTSs (both as binary variables). A clear relationship was found between these two variables: Clusters 1 and 2 showed high shares of car ownership (>90%) and relatively low shares of PTS (Cluster 1: 62%; Cluster 2: 55%). Clusters 3 and 4 indicated lower shares of car ownership but higher shares of PTS (~80%). More than half the respondents in Cluster 2 owned homes; the respondents from Clusters 1 and 3 indicated lower values (46%). The Cluster 4 respondents reported the lowest value by far, at 21%. Clusters 1 and 2 comprised more male respondents, whereas Clusters 3 and 4 showed over 50% females. In Cluster 1, 24 households (17%) earned more than 14,000 Swiss francs (approximately USD 14,500) in net income per month. For the respondents in the most sufficient Cluster 3, this rate was only 6%.

#### 3.2.3 Dependent variables

Table 6 shows the results of an ANOVA testing for differences among the clusters for the dependent variables. Except for one variable (working at home, F_(3, 585)_ = 1.06, *p* = 0.365), all differences were significant (detailed results with Bonferroni multiple comparisons, 99% interval; see the superscript letters in Table 6). Cluster 1 shows the highest values (i.e., nonsufficient ratings compared to the other clusters) for all items except working at home (which was not significantly different). Particularly for the living area, washing, room temperature, and vacation factors, the values in Cluster 1 were considerably higher than those of all the other clusters. In contrast, Cluster 3 showed the lowest ratings of all clusters for all items, except intermittent ventilation. Cluster 3 had particularly low mean values for meat consumption, room temperature, and showering. In other words, its respondents demonstrated comparatively more sufficient activity levels (see Table 4) and consented more readily to the sufficiency scenarios the characters conveyed. Compared to the independent variables, where Cluster 2 was similar to Cluster 1 for some items (e.g., meat consumption and ownership of cars and tools), for the dependent variables, Cluster 2 showed lower (i.e., more sufficient) values, particularly for living area, room temperature, and washing. Based on their current fairly high standard of living and level of behavior, the Cluster 2 respondents may have been more receptive to more sufficient behavioral changes than the Cluster 1 respondents. Cluster 3 participants, identified as having fairly sufficient behavior/activity levels, showed relatively high acceptance of the presented scenarios, particularly for the variables that loaded on Factor 2 (activities at home) as well as for the scenarios related to tool and car sharing. All clusters indicated high values for vacationing in faraway countries. This scenario was thus the least acceptable, followed by working at home (where no significant differences appeared).

**Table 6:** Results of the cluster analysis of the dependent variables. The variables are sorted according to the factors presented in Section 3.1.

Factor	Variable	Cluster 1: Homeowners with car affinity (*N* = 156)^a^		Cluster 2: High standard of living but conscious consumption (*N* = 89)^b^		Cluster 3: Sufficient consumption (*N* = 149)^c^		Cluster 4: Middle group with potential (*N* = 208)^d^	
		Mean	SD	Mean	SD	Mean	SD	Mean	SD
No factor	Vacation	5.1^c,d^	2.07	4.4^c^	2.16	2.8^a,b,d^	2.05	3.9^a,c^	2.30
1	Living area	4.5^a,c,d^	2.15	3.6^a,c^	2.06	2.7^a,b,d^	1.78	3.8^a,c^	2.10
1	Sharing car	3.9^c,d^	2.20	3.4^c,d^	1.92	2.0^a,b^	1.51	2.1^a,b^	1.51
1	Meat consumption	4.0^c^	2.12	3.4	1.98	2.3^a,d^	1.60	3.1^c^	1.90
1	Sharing tools	3.1^c,d^	1.93	2.7^c^	1.66	1.9^a,b^	1.19	2.4^a^	1.61
2	Room temperature	4.1^b,c,d^	2.04	2.7^a,c^	1.98	1.8^a,b,d^	1.34	2.7^a,c^	1.85
2	Showering	3.5^c^	1.97	3.0	1.61	2.3^a,d^	1.56	3.2^c^	1.86
2	Switching off lights	2.6^c,d^	1.97	2.0	1.44	1.6^a^	1.04	2.1^a^	1.45
2	Regional products	2.5^c^	1.52	2.3^c^	1.54	1.7^a,b^	1.00	2.1	1.36
2	Intermittent ventilation	1.9^d^	1.48	1.4	0.98	1.6	1.41	1.5^a^	1.02
3	Washing	3.9^b,c,d^	2.15	2.4^a^	1.78	2.1^a,d^	1.54	3.0^a,c^	1.99
4	Working at home (ns)	3.5	2.12	3.6	2.26	3.5	1.93	3.8	2.02

The higher the mean values, the more nonsufficient the respective behaviors. All differences are significant (*p* < 0.001), except working at home (ns). The superscript letters (a,b,c,d) indicate significant differences among particular clusters (ranging from a = Cluster 1 to d = Cluster 4), *p* < 0.01, using the Bonferroni post-hoc test.

To summarize, we may briefly describe the four clusters as follows: Clusters 1 and 2 showed a fairly high standard of living, with high rates of car and home ownership and relatively high monthly incomes. Notably, more than half the households in Cluster 2 owned their own houses. They also had the highest percentage of children at home and most often belonged to one or more association. The Cluster 3 respondents were the oldest, and they earned less than those in the other clusters; only half owned cars, but they had the same home-ownership percentage as Cluster 1. Cluster 4 comprised the highest number of females of all clusters, with a lower mean age, fewer children, and relatively few car owners.

## 4 Discussion

### 4.1 Summary and discussion of findings

This study investigated the status quo of the need for energy-related behavior and the potential for behavioral change toward increased energy sufficiency. Based on the results, we will now revisit the two questions posed in the introduction. We then discuss some of the limitations of the study and suggest points for future research. In the next two subsections, we answer the first questions: “From an individual perspective, where does the potential exist for behavioral changes via cities’ interventions? Could any thematic sets of behaviors be addressed by an intervention?”

#### 4.1.1 Overall potential

We did find differences in the potential for straightforward behavioral changes, primarily in the section of “easy” behavior presented in Table 2. When analyzing households’ current energy-saving activities, it became clear that certain activities are easier to adopt than others; some of these actions have been proposed since the 1970s [49]. In particular, using intermittent ventilation and switching off lights when leaving a room can be assumed to be current practices in Switzerland, because people are aware of these practices through several campaigns (although actual activities may deviate from self-reported behavior). Table 2 shows a gradient from these rather easy activities (e.g., using intermittent ventilation, switching off lights, and purchasing regional products) to more demanding changes, such as living in reduced living spaces [50, 51]. This finding reveals little potential for improvement in these activities other than their relatively low impact on decreasing energy consumption [23].

In contrast, changing one’s patterns for vacation and living space may mean higher reductions of energy consumption and greenhouse-gas emissions, but these changes are also more difficult to accomplish. This study’s participants seemed to highly value vacation and large living spaces and consequently indicated a tendency toward the more nonsufficient options. A city’s short-term intervention could hardly achieve the considerable energy-saving potential of these cases; such potentials would instead require long-term interventions. We suggest that more general lifestyle changes would have to address these activities in order to become more sufficient in the future [52]. Lifestyle changes are possible, particularly if other life events occur, (such as moving to another place, [53]). One other reason for behavioral inertia, however, may partly be seen in events such as the Paris Agreement on Climate Change (see introduction); some citizens may be misled into thinking things will turn out fine anyway and that their own households’ energy-saving efforts are sufficient, with no further action required.

#### 4.1.2 Thematic sets of behavior

We also found thematic links between certain activities (see the results of the factor analysis), as follows: those pursued at home (daily behavior representing easy activities); those related to the workplace; those concerning hygienic standards; and—the most important in terms of explained variance—those linked to sharing (of cars, tools, and living space). Thus, the four factors suggest that these different domains should be considered when addressing behavioral-energy-consumption changes. Recent research has found similar types of behavior. For instance, Barr et al. [54] found different types of waste behavior, while Barr and Gilg [34] investigated four types of sustainable behavior: green consumption, energy saving, water conservation, and recycling. In their study of consumers’ willingness to address climate change in their actions, Tobler et al. [51] found a three-cluster solution, distinguishing among easy behaviors (e.g., intermittent ventilation), indirect behaviors (e.g., financially offsetting carbon-dioxide emissions from airplanes), and mobility-related activities (e.g., avoiding car use). In addition, according to Dietz and colleagues, “plasticity is probably behavior-specific in ways not fully understood at present” ([52], p.18454). The results of our factor analysis showed Factors 3 and 4 as context-dependent; not everybody can (or would like to) work at home, and the frequency of washing one’s clothes depends on norms and notions of hygiene and whether a household has a washing machine. We could suggest addressing thematically linked behaviors in different ways. Thus, interventions in this case may have to consider these constraints and customize campaigns in adequate ways—for instance, not only by promoting working at home among citizens but also by addressing employers’ awareness of resource use. In our study, we did not find (as Poortinga et al. did in their study [24]) that people generally prefer energy-saving methods at home (measured here with reduced room temperature and switching off lights) to methods that affect mobility (car sharing); see Table 2.

#### 4.1.3 Group differences

We found differences among groups of participants regarding the second questions: “Can different groups of individuals be classified by their current behaviors/activities, conceptualized as independent variables? Are there any links between these behaviors and future behavioral-change potentials, conceptualized as dependent variables?” The respondents in the analyzed clusters differed in their current lifestyles as well as in their potentials for future change. For instance, the Cluster 3 (sufficient consumption) participants already showed a more sufficient current behavioral pattern and were also more willing to change their behaviors, particularly regarding car ownership. The reduction of meat consumption was not problematic for Cluster 3 but was for Cluster 1 (homeowners with car affinity). This was because of the Cluster 1 members’ high standard of living and greater reluctance to make changes (concerning current behaviors/activities *and* behavioral-change potential) compared to other clusters’ members. This finding was particularly true with sharing activities; the ownership of tools and cars was important and unlikely to change easily.

The Cluster 1 respondents also showed fairly high values for living areas and heating, bathing, and taking long showers. Except for working at home—which was not significantly different among the clusters in either dependent or independent variables—Cluster 1 ranked as the most unsustainable for all scenario items. Cluster 2 (high standard of living but conscious consumption) also showed a fairly high behavior/activity level; they reported constraints regarding vacation, living areas, and appliances in standby mode to be most problematic. The ratings of Cluster 4 (middle group with potential) fell between the other clusters, with no distinctive pattern emerging. Nevertheless, this cluster had more participants than the other clusters and did show some potential for behavioral change. Participants generally expressed a desire to own their own tools and household items, although, depending on the context, specific options could make sharing possible. A clear pattern emerged around the clusters nevertheless: Clusters 1 and 2 showed less preference for sharing tools and cars than Clusters 3 and 4, which suggests that the former groups are probably not ready for a sharing economy [55].

Thus, different groups of inhabitants (or households) may currently demonstrate diverse levels of energy-saving behavior in various aspects of daily life and may be willing to change these behaviors to varying degrees. In other words, discernible social groups may show different levels of behavioral plasticity. For instance, Dietz et al. [52] determined from the relevant literature the notion that carpooling and “trip-chaining” (i.e., combining errands) might be the least adopted among the public compared to all other measures they included in the study, but the authors made no differentiation with respect to groups. Based on our results, Clusters 1 and 2 certainly matched this assumption, but we did observe the potential for change in Clusters 3 and 4.

### 4.2 Forms of interventions

For several reasons, our collaborating cities had a special interest in formal social groups as potential addressees of tailored campaigns and interventions [56]. For example, even if an intervention could address specific groups of individuals and behavioral classes, how could people avoid being indifferent and reverting to their previous behavior after a campaign’s termination? While people are often open to energy-saving measures and economic arguments regarding energy efficiency, they are also often not fully convinced that their share is important and will mitigate global climate change, reasoning that their efforts would be a “drop in the ocean.” The belief in the individual’s importance in terms of global problems is actually weak, and changes to one’s own lifestyle are generally viewed as having a low impact [57].

Another problem may be found in potential reactance effects, a phenomenon that arises when others aim to restrict one’s behavior [58]. Thus, it would seem worthwhile to find new ways or channels for interventions [59] and to avoid low commitment because of people’s skepticism about the effects. As Sütterlin ([60], p.22) states, “people differ in their social value orientations (i.e., whether collective long-term interests or personal short-term self-interests prevail).” This difference could be linked to people’s engagement in formal social groups, which is an issue that could be addressed by interventions that focus on social groups such as associations (e.g., sports clubs).

As observed from our sample, all clusters showed a relatively high percentage of membership in one or more associations. Overall, around 40% of Swiss people are active members of associations [61]. In cases where people meet and interact, some sort of mutual social influence [62] may ensure the establishment of long-term traits, thus avoiding the problem of short-term effects. The feeling of being overwhelmed could be countered as well, because a person is obviously not alone in an association. Studies have found clear indications that social capital may be important for pro-environmental behavior; Macias and Williams [63], for example, have investigated (among other variables) activities similar to those examined in the present study. They found that spending evenings with neighbors is a strong predictor of peer influence for four of the six variables they tested, including water conservation, energy conservation, and driving less, among others. Sütterlin et al. ([22], p.8148) likewise proposed information-providing events to “provide an optimal opportunity to sensitize consumers to the implementation of regulatory interventions aiming to reduce energy consumption. Thereby, it is important to emphasize the value of each individual’s contributions and collective thinking. Such events are an ideal setting to establish corresponding social norms.” For workplace groups, information sessions could also be an interesting avenue for interventions, because “the importance of community in the workplace and the role of social norms forming within those groups” appear considerable ([64], p.39).

For cities, this point offers a hint that campaigns that contain a social component may be a valuable option for intervention. Research on the social-identity model [65] has found that identifying with a group is important for collective action in several ways, both directly and through connections with the sense of injustice and considerations of a group’s efficacy. In this regard, a group may be perceived as more effective than an individual. Social identity, as mentioned in the introduction, could thus be a strong component of interventions, because “a stronger sense of social identity should relate to a stronger motivation to engage in collective action (through stronger adherence to group norms), a stronger perception and experience of injustice (through group based emotional experience), and a stronger sense of efficacy (through empowerment)” ([65], p.524).

Addressing energy-saving activities indirectly via associations, without direct imposition by cities, may be a proper way to include citizens in energy-system transitions. Taking the social environment into account offers the opportunity to indirectly influence individuals. For instance, associations and other formal social groups may provide the necessary direct experience by sharing experiences and opinions in Tupperware party—like settings [66]. Social comparison is one element that affects environmental- and energy-related behavior [67, 68]. Exchanges about knowledge and experiences in groups may thus influence the members’ energy behavior. Knowing what others think and do with respect to an energy-consumption-reduction campaign and related behavioral options, for example, has been linked to social comparison. For instance, Rose and Marfurt [69] showed this link in a “Ride to Work Day” campaign for bicycle use.

In addition, no single authority prescribes curtailment norms (which would lead to reactance, but people generally consider these norms through social comparison and their need for social acceptance by identifying with energy-saving group norms. The avoidance of reactance therefore seems possible when positive experiences in a familiar group indicate new ways of reducing energy consumption without focusing on guilt or obligation. A recent study evaluated an intervention that offered free e-bike trials to motivate Swiss car owners to try out new mobility patterns; the participants’ intention to change their mobility-related behaviors to more sufficient ones correlated with the positive feedback they received from friends and family members [70]. The present study’s results demonstrated groups of participants’ (clusters’) differences regarding their engagement in associations. Together with the behavioral pattern, this information may be helpful when addressing these clusters in campaigns or interventions.

### 4.3 Limitations of the study

One limitation of this study is its cross-sectional design. It would be preferable to use data on the same cohort using a longitudinal design to measure real change in the participants’ behavior. We were able to assess the current state of their behaviors/activities, however (and thus the degree of sufficiency); we also related this sufficiency to future reduction potential vis-à-vis energy-consumption behavior.

The sample may also appear nonoptimal, since we worked with cities that already actively promoted energy-demand reduction among their citizens; the results thus may have limited transferability to other cities that are currently not as active in their energy-consumption-reduction campaigns. The results’ transferability to the rural population may also be restricted due to differences in exposure to behavioral interventions in the energy sector as well as their personal life situations, such as car ownership and use, home ownership, and workplace circumstances.

We did not include specific measures for environmental attitudes, although it could be interesting to determine whether the clusters’ respective members would differ in their opinions on the “new ecological paradigm” items [71], for instance. In addition, the independent and dependent variables were fairly similar in our study, although the former were intended as measures for current behaviors and needs, whereas the latter were meant to estimate potential behavioral change. It may be that the correlation between both sets of variables somewhat distorted the results; nevertheless, the clusters were distinctive in a nontrivial way.

### 4.4 Suggestions for further research

Referring to stage models [35], different groups of people can be assumed to be at different phases of intention development and actual behavior. How and when certain interventions leverage behavioral intentions may thus differ, which cities must consider when planning interventions. In our study, we did not fully capture the respondents’ respective stages, which would have enabled us to link our groups to Bamberg’s stage concept [35], for example. Based on the results, however, we can provide a few tentative insights into this matter that clearly demonstrate a valuable direction for future research.

We have shown that at the time of our survey, some clusters of participants had already reached more sufficient levels of activity than others, and they even indicated more willingness for further change within specific groups of activities. Given the differences in the clusters’ basic willingness to adopt energy-saving behavior, variations may also exist in responses to different intervention types. For instance, because Cluster 3 participants already reported low meat consumption and were open to sharing tools and cars, a high level of environmental awareness, social norms, and goal-related, behavioral, and implementation intentions could be assumed among that cluster. In contrast, the Cluster 1 members did not display adequate levels of activity and lacked sufficiency behavior with respect to vacation destinations, living areas, room temperatures, and sharing. In this case, we may assume that social norms and other factors would prevent proper goal intentions.

## 5 Conclusions

This study’s results indicate that groups of participants show certain current behaviors as well as distinctive potentials for future behavioral change. The type of behavior is important. The medium group of activities (which are neither trivial nor too extreme) may generally offer the greatest potential for change while contributing significantly to the reduction of energy consumption and greenhouse-gas emissions. These activities include sharing tool kits, rooms, and cars; maintaining lower room temperatures; washing less frequently and taking shorter showers; and consuming less meat. These types of activities may depend on different settings or contexts, however, and they may show specific patterns for different subgroups. For instance, sharing things differs from working at home and washing as well as from daily behavior at home, such as maintaining lower room temperatures and taking shorter showers. In particular, the two factors—washing and working at home—may be linked to specific lifestyles and contexts.

When addressing citizens’ sufficiency behaviors, administrations should be aware that people are not homogeneous and may be at different stages of intention development regarding their energy-saving behavior. Tailored information and interventions that address different subgroups in specific ways could help achieve success with these activities. Our cluster analysis clearly points in this direction, although further research is necessary to gain a better understanding of how interventions may be tailored toward these groups. New studies are required to investigate whether different empirical groups (such as this study’s clusters) are linked to various stages.

What does this finding mean for cities’ planned future interventions? Currently, easy behaviors—such as taking short showers, bathing infrequently, putting devices on standby mode, and consuming goods that are produced locally—do not rank high on the list of behaviors/activities. On the contrary, people’s ownership of spacious living areas and their desire to own their own tools and cars are more important (and hence presumably harder to change). It might be difficult, however, to precisely target these behaviors without eliciting reactance, especially in comparatively wealthy countries such as Switzerland, where some citizens, due to their high income, are used to a resource-intensive lifestyle and hope to maintain their high living standards (see our Clusters 1 and 2). But it would be interesting to examine the reasons behind the purported need for large living areas, for instance. Options such as shared rooms would likely be acceptable if they were communicated with a tailored approach to the different groups that considered their respective contexts. Further studies are necessary to investigate the needs behind different groups’ energy-consumption behaviors.

## Supporting information

S1 File Survey—Original German version. (PDF)

S2 File Survey—English translation. (PDF)
