# Supplementary material for: Navigating behavioral energy sufficiency. Results from a survey in Swiss cities on potential behavior change
Source: PLoS One. 2017 Oct 9;12(10):e0185963. doi: 10.1371/journal.pone.0185963 (PMC5633184; doi:10.1371/journal.pone.0185963)
Supplement: S1 File — (PDF) [file pone.0185963.s001.pdf]

Druckversion

Survey – original German version

Fragebogen

1 Start

Herzlich Willkommen zur Umfrage „aktive Zukunftsgestaltung“!

Mit diesem Fragebogen wollen wir herausfinden, wie die Winterthurer Bevölkerung zu Veränderungen in verschiedenen Lebensbereichen steht.
Ihre Antworten werden anonym ausgewertet, nur für Forschungszwecke verwendet und nicht an Dritte weitergegeben.
Wenn Sie Fragen zur Umfrage haben, können Sie sich gerne an Frau Dr. ----- wenden.
Vielen Dank für Ihre Unterstützung!

2 PLZ

Wie lautet die Postleitzahl Ihres Wohnortes?

3.1 Ausgescreent

4 Geschlecht

Was ist Ihr Geschlecht?

- männlich
- weiblich

5.1 Filter

6 Bedürfnisse

Im ersten Teil der Umfrage interessiert uns, was Ihnen im alltäglichen Leben wichtig ist.

Bitte tragen Sie die Wichtigkeit auf den Skalen von 1 (gar nicht wichtig) bis 7 (sehr wichtig) ein.

Wie wichtig ist Ihnen...

	1 (gar nicht wichtig)	2	3	4	5	6	7 (sehr wichtig)
...viel Wohnfläche zur Verfügung zu haben?							
...dass es in Ihrer Wohnung im Winter auch ohne Pullover gemütlich warm ist?							
...im Winter mit geöffnetem Fenster zu schlafen?							
...Ihre tägliche Tätigkeit (Erwerbstätigkeit, Studium, Freiwilligenarbeit, usw.) am Arbeitsplatz und nicht zu Hause durchzuführen?							
...in fernen Ländern Ferien zu machen?							
...in der Nähe Ihres Arbeits- bzw. Studienortes zu wohnen?							
...eigene Werkzeuge bzw. Haushaltsgeräte zu besitzen?							
...dass Ihre elektronischen Geräte komplett ausgeschaltet sind, wenn sie nicht benutzt werden?							
...ein eigenes Auto zu besitzen?							
...regional produzierte Produkte zu kaufen?							
...mehrmals wöchentlich zu baden?							
...Fleischkonsum?							
...täglich frisch gewaschene Kleider zu tragen? (z.B. Pullover; nicht gemeint sind hier Unterwäsche und Socken)							
...lange zu duschen?							

7 Zweiter Teil

Im nächsten Teil der Umfrage sind die Fragestellungen rund um die beiden fiktiven Personen Toni und Mira sowie deren Umfeld aufgebaut. Bei den ersten acht Fragen haben Toni und Mira unterschiedliche Ansichten. Hier interessiert uns, mit wem Sie sich eher identifizieren können.

Weitere fünf Fragen beinhalten Szenarien, bei denen Mira und Toni gleicher Meinung sind. Hier interessiert uns, wie attraktiv diese Szenarien für Sie sind.

8 Raumtemperatur V1

Mira ist der Meinung, dass die Wohnung im Winter nur so fest geheizt werden muss, dass sie sich mit einem Pullover wohl fühlt. Toni ist mit Mira gar nicht einverstanden und will es so warm haben, dass ein T-Shirt ausreicht.

Mit wem können Sie sich eher identifizieren?

Mira ○ ○ ○ ○ ○ ○ ○ Toni

Ihre Bemerkungen dazu:

9 Home Office

Im Einvernehmen mit seinem Arbeitgeber erledigt Toni manchmal seine Arbeit von Zuhause aus. Für Mira ist das nichts, obwohl sie auch die Möglichkeit dazu hätte. Sie verlässt lieber das Haus, um zu arbeiten.

Mit wem können Sie sich eher identifizieren?

Mira ○ ○ ○ ○ ○ ○ ○ Toni

Ihre Bemerkungen dazu:

10 Arbeitsweg

Mira schätzt es, in unmittelbarer Umgebung ihres Wohnortes zu arbeiten, damit sie bei schönem Wetter zu Fuss zur Arbeit gehen kann. Toni macht das Pendeln nichts aus, wenn seine Arbeitsstelle seinen Wünschen entspricht. Er ist daher gerne bereit, einen längeren Arbeitsweg auf sich zu nehmen.

Mit wem können Sie sich eher identifizieren?

Toni ○ ○ ○ ○ ○ ○ ○ Mira

Ihre Bemerkungen dazu:

11 Waschen

Toni trägt denselben Pullover während mehreren Tagen. Mira bevorzugt es, jeden Tag einen frisch gewaschenen Pullover zu tragen.

Mit wem können Sie sich eher identifizieren?

Toni ○ ○ ○ ○ ○ ○ ○ Mira

Ihre Bemerkungen dazu:

12 Stromnutzung

Wenn möglich, löscht Toni das Licht. Ihm ist nur wichtig, dass dort genügend Licht vorhanden ist, wo er sich gerade befindet. Mira fühlt sich mit viel Licht in der Wohnung wohl und lässt die Beleuchtung jeweils brennen bis sie schlafen geht oder die Wohnung verlässt.

Mit wem können Sie sich eher identifizieren?

Toni ○ ○ ○ ○ ○ ○ ○ Mira

Ihre Bemerkungen dazu:

13 Warmwasser

Toni und Mira haben einen Duschkopf mit verschiedenen Einstellmöglichkeiten. Während Mira die Wassersparfunktion benutzt, bevorzugt Toni die Massagefunktion.

Mit wem können Sie sich eher identifizieren?

Mira ○ ○ ○ ○ ○ ○ ○ Toni

Ihre Bemerkungen dazu:

14 Fleischkonsum

Mira liebt Fleisch und gönnt sich dies auch täglich. Toni isst auch gerne Fleisch, jedoch nur einmal pro Woche.

Mit wem können Sie sich eher identifizieren?

Toni ○ ○ ○ ○ ○ ○ ○ Mira

Ihre Bemerkungen dazu:

15 Ferien

Toni und Mira planen eine Woche Ferien in Berlin. Toni schlägt vor zu fliegen. Mira will mit dem Nachtzug fahren.

Mit wem können Sie sich eher identifizieren?

Toni ○ ○ ○ ○ ○ ○ ○ Mira

Ihre Bemerkungen dazu:

16 Raumtemperatur V2

Toni ist der Meinung, dass die Wohnung im Winter nur so fest geheizt werden muss, dass er sich mit einem Pullover wohl fühlt. Mira ist mit Toni gar nicht einverstanden und will es so warm haben, dass ein T-Shirt ausreicht.

Mit wem können Sie sich eher identifizieren?

Toni ○ ○ ○ ○ ○ ○ ○ Mira

Ihre Bemerkungen dazu:

17 Home Office

Im Einvernehmen mit ihrem Arbeitgeber erledigt Mira manchmal ihre Arbeit von Zuhause aus. Für Toni ist das nichts, obwohl er auch die Möglichkeit dazu hätte. Er verlässt lieber das Haus, um zu arbeiten.

Mit wem können Sie sich eher identifizieren?

Toni ○ ○ ○ ○ ○ ○ ○ Mira

Ihre Bemerkungen dazu:

18 Arbeitsweg

Toni schätzt es, in unmittelbarer Umgebung seines Wohnortes zu arbeiten, damit er bei schönem Wetter zu Fuss zur Arbeit gehen kann. Mira macht das Pendeln nichts aus, wenn ihre Arbeitsstelle ihren Wünschen entspricht. Sie ist daher gerne bereit, einen längeren Arbeitsweg auf sich zu nehmen.

Mit wem können Sie sich eher identifizieren?

Mira ○ ○ ○ ○ ○ ○ ○ Toni

Ihre Bemerkungen dazu:

19 Waschen

Mira trägt denselben Pullover während mehreren Tagen. Toni bevorzugt es, jeden Tag einen frisch gewaschenen Pullover zu tragen.

Mit wem können Sie sich eher identifizieren?

Mira ○ ○ ○ ○ ○ ○ ○ Toni

Ihre Bemerkungen dazu:

20 Stromnutzung

Wenn möglich, löscht Mira das Licht. Ihr ist nur wichtig, dass dort genügend Licht vorhanden ist, wo sie sich gerade befindet. Toni fühlt sich mit viel Licht in der Wohnung wohl und lässt die Beleuchtung jeweils brennen bis er schlafen geht oder die Wohnung verlässt.

Mit wem können Sie sich eher identifizieren?

Mira ○ ○ ○ ○ ○ ○ ○ Toni

Ihre Bemerkungen dazu:

21 Warmwasser

Mira und Toni haben einen Duschkopf mit verschiedenen Einstellmöglichkeiten. Während Toni die Wassersparfunktion benutzt, bevorzugt Mira die Massagefunktion.

Mit wem können Sie sich eher identifizieren?

Toni ○ ○ ○ ○ ○ ○ ○ Mira

Ihre Bemerkungen dazu:

22 Fleischkonsum

Toni liebt Fleisch und gönnt sich dies auch täglich. Mira isst auch gerne Fleisch, jedoch nur einmal pro Woche.

Mit wem können Sie sich eher identifizieren?

Mira ○ ○ ○ ○ ○ ○ ○ Toni

Ihre Bemerkungen dazu:

23 Ferien

Toni und Mira planen eine Woche Ferien in Berlin. Mira schlägt vor zu fliegen. Toni will mit dem Nachtzug fahren.

Mit wem können Sie sich eher identifizieren?

Mira ○ ○ ○ ○ ○ ○ ○ Toni

Ihre Bemerkungen dazu:

24 Lüften

Mira und Toni stören sich an stickiger Luft. Um während der Heizperiode möglichst wenig Wärme zu verlieren, lüften sie gelegentlich kurz und kräftig, anstelle eines ständig geöffneten Kippfensters.

Ist dieses Szenario attraktiv für Sie?

gar nicht attraktiv ○ ○ ○ ○ ○ ○ ○ sehr attraktiv

Ihre Bemerkungen dazu:

25 Wohnfläche

Toni und Mira haben sich nach einer neuen Wohnung umgeschaut. Sie haben ein Angebot angenommen, bei dem die Wohnung kleiner als die bisherige ist. Dafür können sie bei Bedarf Gästezimmer und Aufenthaltsräume im Gebäude nutzen.

Ist dieses Szenario attraktiv für Sie?

gar nicht attraktiv ○ ○ ○ ○ ○ ○ ○ sehr attraktiv

Ihre Bemerkungen dazu:

26 Regionale Produkte

Toni und Mira kaufen bevorzugt regionale Produkte. Falls diese nicht erhältlich sind, kaufen sie möglichst nachhaltig produzierte Importware.

Ist dieses Szenario attraktiv für Sie?

gar nicht attraktiv ○ ○ ○ ○ ○ ○ ○ sehr attraktiv

Ihre Bemerkungen dazu:

27 Werkzeuge und Haushaltsgeräte

Mira und Toni besitzen einen Veloanhänger für grössere Einkäufe. Nachbar Herbert besitzt einen Akku-Bohrer. Nachbarin Franziska im zweiten Stock besitzt ein Zelt. Da alle drei Gegenstände unregelmässig genutzt werden und die nachbarschaftlichen Beziehungen sehr gut sind, haben sich die vier entschieden, diese Gegenstände einander auszuleihen.

Ist dieses Szenario attraktiv für Sie?

gar nicht attraktiv ○ ○ ○ ○ ○ ○ ○ sehr attraktiv

Ihre Bemerkungen dazu:

28 Car Sharing

Mira und Toni haben ihr Auto verkauft als sie umgezogen sind. Trotzdem benötigen sie ab und zu ein Auto. Nachbar Herbert stellt ihnen bei Bedarf sein Auto zur Verfügung und erhält dafür eine Entschädigung. Sowohl Herbert wie auch Toni und Mira sparen dadurch Geld.

Ist dieses Szenario attraktiv für Sie?

gar nicht attraktiv ○ ○ ○ ○ ○ ○ ○ sehr attraktiv

Ihre Bemerkungen dazu:

29 Dritter Teil

Im dritten Teil der Umfrage geht es um die Kommunikation von Energiethemen.

Mira und Toni sind neu nach Winterthur gezogen. Sie möchten wissen, wie sie sich über Angebote und Veranstaltungen in Winterthur zu Energie und Energiesparen informieren können. Was empfehlen Sie den beiden?

Stichworte genügen:

30 Fragen zu Stadt

Von welchen der folgenden städtischen Aktivitäten im Bereich Energie haben Sie schon gehört oder gelesen?

Bitte alle zutreffenden Antworten ankreuzen:

- ☐ Förderprogramm Energie Winterthur
- ☐ Energieberatung
- ☐ Energiestadt Gold Winterthur
- ☐ Wir leben 2000 Watt
- ☐ KlimaLandsgemeinde
- ☐ Luftaus.ch
- ☐ Umweltbericht Stadt Winterthur
- ☐ Energie- und Umweltapéro
- ☐ energyday
- ☐ Tag der Sonne

weitere: ____

Welches sind für Sie die wichtigsten Informationsquellen zu den Themen Energie und Energiesparen?

Bitte wählen Sie maximal 5 Quellen (durch auswählen und verschieben) und ordnen Sie diese nach Wichtigkeit (wichtigste Quelle zuoberst)

	1	2	3	4	5	6	7	8	9	10	11	12
Websuche (z.B. Google, Wikipedia)	○	○	○	○	○	○	○	○	○	○	○	○
soziale Medien (z.B. Twitter, Facebook)	○	○	○	○	○	○	○	○	○	○	○	○
Newsletter	○	○	○	○	○	○	○	○	○	○	○	○
Zeitungen und Zeitschriften	○	○	○	○	○	○	○	○	○	○	○	○
Fernsehen	○	○	○	○	○	○	○	○	○	○	○	○
Freunde, Familie	○	○	○	○	○	○	○	○	○	○	○	○
Bundesamt für Energie (z.B. energieschweiz)	○	○	○	○	○	○	○	○	○	○	○	○
Nachbar/innen	○	○	○	○	○	○	○	○	○	○	○	○
Arbeits- oder Studienkolleg/innen	○	○	○	○	○	○	○	○	○	○	○	○
Messen und Ausstellungen	○	○	○	○	○	○	○	○	○	○	○	○
Umweltorganisationen	○	○	○	○	○	○	○	○	○	○	○	○
Vorträge	○	○	○	○	○	○	○	○	○	○	○	○

Sonstige Informationsquellen:

31 Sprechen über Energiesparen

Mit wem haben Sie im letzten halben Jahr über die Themen Energie und Energiesparen gesprochen?

Bitte alle zutreffenden Antworten ankreuzen:

- ☐ Freund/innen und Bekannte
- ☐ Arbeits- bzw. Studienkolleg/innen
- ☐ Familie
- ☐ Personen aus der Stadtverwaltung Winterthur (z.B. Energieberatung)
- ☐ Personen vom Stadtwerk Winterthur
- ☐ Personen aus meinem Verein
- ☐ Hauswart/in
- ☐ Hausverwaltung
- ☐ Nachbar/innen
- ☐ mit niemandem

Wenn es um die Themen Energie und Energiesparen geht, wie stark vertrauen Sie den Informationen von folgenden Personen und Institutionen?

Bitte geben Sie auf der Skala von 1 (gar kein Vertrauen) bis 7 (sehr grosses Vertrauen) an, wie stark Ihr Vertrauen ist.

	1 (gar kein Vertrauen)	2	3	4	5	6	7 (sehr grosses Vertrauen)
Freund/innen und Bekannten	○	○	○	○	○	○	○
Familie	○	○	○	○	○	○	○
Bundesamt für Energie	○	○	○	○	○	○	○
Stadtwerk Winterthur	○	○	○	○	○	○	○
Energiefachstelle der Stadt Winterthur	○	○	○	○	○	○	○
Wissenschaft	○	○	○	○	○	○	○
Umweltorganisationen	○	○	○	○	○	○	○
Arbeits- bzw. Studienkolleg/innen	○	○	○	○	○	○	○
Hausverwaltung	○	○	○	○	○	○	○
Hauswart/in	○	○	○	○	○	○	○

32 Fragen zur heutigen Situation

Zum Schluss der Umfrage werden Ihnen noch einige Fragen zu Ihrer Person und Ihrer aktuellen Lebenssituation gestellt.

Sind Sie im Besitz der Liegenschaft bzw. der Wohnung, in der Sie leben?

- ○ ja
- ○ nein

Wie viele Personen leben in Ihrem Haushalt (Sie mit eingeschlossen)?

1
2
3
4
5
6
7
8
9
> 9

Leben minderjährige Personen in Ihrem Haushalt?

- ○ ja
- ○ nein

Welche Grössenordnung hat Ihre Wohnung bzw. Ihr Haus (ohne Estrich, Keller, Balkon)?

-- weiss ich nicht --
< 31 m2
31-40 m2
41-50 m2
51-60 m2
61-70 m2
71-80 m2
81-90 m2
91-100 m2
101-110 m2
111-120 m2
121-130 m2
131-140 m2
141-150 m2
151-160 m2
161-170 m2
171-180 m2
> 180 m2

Welches Stromprodukt beziehen Sie zu Hause?

- e-Strom.Gold
- e-Strom.Silber
- e-Strom.Bronze
- e-Strom.Weiss
- e-Strom.Grau
- weiss ich nicht
- anderes

Welches Verkehrsmittel benutzen Sie am häufigsten (nach Anzahl Wege)?

Fahrrad
Elektro-Fahrrad
Mofa
Roller
Motorrad
Auto
öffentlicher Verkehr
-- anderes --

Wie weit ist die Distanz von Ihrem Zuhause zu Ihrem Arbeitsort (bzw. Studienort, usw.)?

Falls Sie die Distanz nicht genau wissen, bitten wir Sie, diese zu schätzen.

- Anzahl Kilometer (bitte nur Zahlen eingeben): ______
- ich pendle nicht

Besitzen Sie oder eine Person in Ihrem Haushalt ein Auto?

- ja
- nein

Besitzen Sie ein Abonnement (ohne Halbtax) für den öffentlichen Verkehr (z.B. GA, ZVV-Abo, usw.)?

- ja
- nein

Wie oft sind Sie im Jahr 2014 für private Zwecke geflogen (hin und zurück gilt als zweimal)?

nie
1 mal
2 mal
3 mal
4 mal
5 mal
6 mal
mehr als 6 mal

An wie vielen Tagen pro Woche essen Sie üblicherweise Fleisch?

nie
< 1
1
2
3
4
5
6
7

Wie oft kaufen/beziehen Sie ein neues Mobiltelefon?

-- ich besitze kein Mobiltelefon --
mehr als einmal im Jahr
einmal im Jahr
jedes zweite Jahr
jedes dritte Jahr
jedes vierte Jahr
weniger als jedes vierte Jahr
mein aktuelles Mobiltelefon ist m

Als wie umweltbewusst schätzen Sie sich ein?

gar nicht umweltbewusst ◯ ◯ ◯ ◯ ◯ ◯ ◯ sehr umweltbewusst

33 Demographische Fragen

In welchem Jahr sind Sie geboren?

1917
1918
1919
1920
1921
1922
1923
1924
1925
1926
1927
1928
1929
1930
1931
1932
1933
1934
1935
1936
1937
1938
1939
1940
1941
1942
1943
1944
1945
1946
1947
1948
1949
1950
1951
1952
1953
1954
1955
1956
1957
1958
1959
1960
1961
1962
1963
1964
1965
1966
1967
1968
1969
1970
1971
1972
1973

Was ist Ihr höchster Bildungsabschluss?

kein Schulabschluss
obligatorische Schule
Anlehre, Haushaltslehrjahr
Berufslehre, Berufsmittelschule,
Berufsmatura, Maturitätsschule,
Meisterprüfung, Techniker- und F
Fachhochschule, Universität, ETH
andere Ausbildung

Was ist Ihr Beruf?

Wie hoch ist das monatliche Nettoeinkommen (Einkommen nach Abzügen) Ihres Haushaltes? (Summe aller Einkommen Ihres Haushaltes)

-- keine Angaben --
< 4'000 SFr.
4'001 - 6000 SFr.
6'001 - 8000 SFr.
8'001 - 10000 SFr.
10'001 - 12'000 SFr.
12'001 - 14'000 SFr.
14'001 - 16'000 SFr.
16'001 - 18'000 SFr.
> 18'000 SFr.

Wie schätzen Sie Ihre politische Haltung ein?

links ○ ○ ○ ○ ○ ○ ○ rechts

Welche politische Partei vertritt Ihre Interessen am ehesten?

-- keine Angaben --
-- keine Partei --
AL - Alternative Liste
CVP - Christlichdemokratische Vo
FDP - Freisinnig-Demokratische P
Grüne / Junge Grüne
SVP - Schweizerische Volkspartei
SP - Sozialdemokratische Partei
Piraten - Piratenpartei
GLP - Grünliberale Partei
EVP - Evangelische Volkspartei
EDU - Eidgenössisch-Demokratis
andere

Sind Sie aktives Mitglied in einem oder mehreren Verein(en)?

Bitte alle zutreffenden Antworten ankreuzen:

- ☐ nein
- ☐ ja, im Bereich Sport
- ☐ ja, im Bereich Musik
- ☐ ja, im Bereich Politik
- ☐ ja, im Bereich Umwelt
- ☐ ja, in einem anderen Bereich

34 Kommentare und Anregungen

Vielen Dank für Ihre Teilnahme!

Klicken Sie bitte auf Weiter, um Ihre intervista Bonuspunkte zu erhalten.

35 Endseite
