# Supplementary material for: Navigating behavioral energy sufficiency. Results from a survey in Swiss cities on potential behavior change
Source: PLoS One. 2017 Oct 9;12(10):e0185963. doi: 10.1371/journal.pone.0185963 (PMC5633184; doi:10.1371/journal.pone.0185963)
Supplement: S2 File — (PDF) [file pone.0185963.s002.pdf]

Survey – English translation

Questionnaire

1 Start

Welcome to the survey "Shaping the future"!

With this questionnaire we want to find out how the population of Winterthur is changing in different areas of life. Your answers will be anonymous, used only for research purposes and not shared with third parties.
If you have any questions regarding the survey, please do not hesitate to contact ---- .
Thank you for your support!

2 ZIP code

What is the zip code of your place of residence?

4 Gender

What is your gender?

- male
- female

5.1 Filter

6 Needs

In the first part of the survey we are interested in what is important to you in everyday life.

Please enter the importance on the scales from 1 (not important at all) to 7 (very important).

How important is it to you...

	1 (not at all important)	2	3	4	5	6	7 (very important)
have a large living area at your disposal?							
be comfortably warm without a sweater during the winter?							
sleep with an open window during the winter?							
perform your daily tasks (work, study, etc.) at the workplace and not at home?							
go on vacation to remote countries?							
live far away from work?							
own tools and household aids?							
put devices on standby mode?							
own a car?							
consume goods that are not produced locally?							
take a bath more than once a week?							
consume meat?							
wear freshly washed clothes (e.g. a sweater, aside from underwear)?							
take a long shower?							

7 Second part

In the next part of the survey the questions deal with two fictitious persons Toni and Mira as well as their environment. In the first eight questions Toni and Mira have different views. Here we are interested with whom you can identify with.

Another five questions include scenarios where Mira and Toni share the same opinion. Here we are interested in how attractive these scenarios are for you.

8 Room temperature

Mira thinks that the apartment should be heated only to a degree that she feels comfortable wearing a pullover. Tony doesn’t agree with Mira at all. He wants to have it warm so that wearing a T-shirt is sufficient.”
With whom can you identify more?

Mira ○ ○ ○ ○ ○ ○ ○ Tony

Your comments on that topic:

9 Working at home

In agreement with his employer, Tony sometimes does his work from home. Mira doesn't like that, although she would have the opportunity to do so. She prefers leaving the house to work.
With whom can you identify more?

Mira ○ ○ ○ ○ ○ ○ ○ Tony

Your comments on that topic:

10 Commute to work

Mira appreciates working in the immediate vicinity of her place of residence so she can walk to work in good weather. Tony does not mind the commuting if his working place meets his wishes. He is therefore willing to take a long journey.

With whom can you identify more?

Tony ○ ○ ○ ○ ○ ○ ○ Mira

Your comments on that topic:

11 Washing

Tony wears the same sweater for several days. Mira prefers wearing a freshly washed sweater every day.

With whom can you identify more?

Tony ○ ○ ○ ○ ○ ○ ○ Mira

Your comments on that topic:

12 Power use

If possible, Tony switches off the light. It is only important to him that there is enough light there, where he is. Mira feels comfortable with a lot of light in the apartment and lets the lighting burn until she goes to sleep or leaves the apartment.

With whom can you identify more?

Tony ○ ○ ○ ○ ○ ○ ○ Mira

Your comments on that topic:

13 Warm water

Tony and Mira have a shower head with various adjustment possibilities. While Mira uses the water-saving function, Tony prefers the massage function.

With whom can you identify more?

Mira ○ ○ ○ ○ ○ ○ ○ Tony

Your comments on that topic:

14 Meat consumption

Mira loves meat and eats it daily. Toni also likes to eat meat, but only once a week.

With whom can you identify more?

Tony ○ ○ ○ ○ ○ ○ ○ Mira

Your comments on that topic:

15 Vacation

Tony and Mira are planning a week holiday in Berlin. Tony suggests to fly. Mira wants to take the night train.

With whom can you identify more?

Tony ○ ○ ○ ○ ○ ○ ○ Mira

Your comments on that topic:

16 Room temperature (2)

Tony thinks that the apartment should be heated only to a degree that he feels comfortable wearing a pullover. Mira doesn't agree with Tony at all. She wants to have it warm so that wearing a T-shirt is sufficient."

With whom can you identify more?

Tony ○ ○ ○ ○ ○ ○ ○ Mira

Your comments on that topic:

17 Working at home

In agreement with her employer, Mira sometimes does her work from home. For Tony that is nothing, although he would have the possibility. He prefers leaving the house to work.

With whom can you identify more?

Tony ○ ○ ○ ○ ○ ○ ○ Mira

Your comments on that topic:

18 Commute to work

Tony appreciates working in the immediate vicinity of his place of residence so that he can walk to work in good weather on foot. Mira does not bother about commuting if her workplace meets their wishes. She is therefore willing to take a long journey.

With whom can you identify more?

Mira ○ ○ ○ ○ ○ ○ ○ Tony

Your comments on that topic:

19 Washing

Tony wears the same sweater for several days. Mira prefers wearing a freshly washed sweater every day

With whom can you identify more?

Mira ○ ○ ○ ○ ○ ○ ○ Tony

Your comments on that topic:

20 Stromnutzung

If possible, Mira switches off the light. It is only important that there is enough light there, where she is. Tony feels comfortable with a lot of light in the apartment and lets the lighting burn in each case until he goes to sleep or leaves the apartment.

With whom can you identify more?

Mira ○ ○ ○ ○ ○ ○ ○ Tony

Your comments on that topic:

21 Warm water

Mira and Tony have a shower head with different settings. While Tony uses the water-saving function, Mira prefers the massage function.

With whom can you identify more?

Tony ○ ○ ○ ○ ○ ○ ○ Mira

Your comments on that topic:

22 Meat consumption

Tony loves meat and eats it daily. Miry also likes to eat meat, but only once a week.

With whom can you identify more?

Mira ○ ○ ○ ○ ○ ○ ○ Ton

Your comments on that topic:

23 Vacation

Tony and Mira are planning a week holiday in Berlin. Mira suggests to fly. Tony wants to take the night train.

With whom can you identify more?

Mira ○ ○ ○ ○ ○ ○ ○ Tony

Your comments on that topic:

24 Airing rooms

Mira and Tony interfere with stuffy air. In order to lose as little heat as possible during the heating period, they occasionally ventilate briefly and vigorously, instead of a constantly opened tilted window.

Is this scenario attractive to you?

not at all attractive ○ ○ ○ ○ ○ ○ ○ very attractive

Your comments on that topic:

25 Living area

Tony und Mira haben sich nach einer neuen Wohnung umgeschaut. Sie haben ein Angebot angenommen, bei dem die Wohnung kleiner als die bisherige ist. Dafür können sie bei Bedarf Gästezimmer und Aufenthaltsräume im Gebäude nutzen.

Is this scenario attractive to you?

not at all attractive ○ ○ ○ ○ ○ ○ ○ very attractive

Your comments on that topic:

26 Regional products

Tony and Mira prefer to buy regional products. If these are not available, they will buy imported products produced as sustainable as possible.

Is this scenario attractive to you?

not at all attractive ○ ○ ○ ○ ○ ○ ○ very attractive

Your comments on that topic:

27 Tools and household appliances

Mira and Tony have a bike trailer for larger purchases. Neighbor Herbert has a battery drill. Neighbor Franziska on the second floor has a tent. Since all three objects are used irregularly and the neighborly relations are very good, the four have decided to lend and borrown these objects among each other.

Is this scenario attractive to you?

not at all attractive ○ ○ ○ ○ ○ ○ ○ very attractive

Your comments on that topic:

28 Car sharing

Mira and Tony have sold their car as they moved. Nevertheless they need a car from time to time. Neighbor Herbert will provide them with his car if they need it and receives a compensation. Both Herbert and Tony and Mira save money.

Is this scenario attractive to you?

not at all attractive ○ ○ ○ ○ ○ ○ ○ very attractive

Your comments on that topic:

29 Third part

The third part of the survey deals with the communication of energy topics.

Mira and Toni have moved to Winterthur. You would like to know how to find out about offers and events in Winterthur about energy and energy saving. What do you recommend the two?

Keywords suffice:

30 Questions regarding your city

Which of the following urban activities in the field of energy have you heard of or read about?

Please tick all that apply:

- ☐ Förderprogramm Energie Winterthur
- ☐ Energieberatung
- ☐ Energiestadt Gold Winterthur
- ☐ Wir leben 2000 Watt
- ☐ KlimaLandsgemeinde
- ☐ Luftaus.ch
- ☐ Umweltbericht Stadt Winterthur
- ☐ Energie- und Umweltapéro
- ☐ energyday
- ☐ Tag der Sonne

additional:

Which hat are the most important sources of information on energy and energy saving?

Please select a maximum of 5 sources (by selecting and moving) and rank them by importance (the most important source at the top)

	1	2	3	4	5	6	7	8	9	10	11	12
Web search (e.g. Google, Wikipedia)												
Social media (e.g., Twitter, Facebook)												
Newsletter												
Newspapers and periodicals												
TV												
Friends, family												
Federal Office of Energy (e.g., energieschweiz)												
Neighbors												
Work or study colleagues												
fairs and exhibitions												
environmental organizatio[illegible]												
Presentations												

Other sources of information:

31 Talking about energy saving

With whom did you talk about energy and energy savings in the last six months?

Please tick all that apply:

- Friends and acquaintances
- Work or study collegues
- Family
- Persons from the town administration of Winterthur (for example energy advice)
- People from the town of Winterthur
- People from my club
- Caretaker
- Property management
- Neighbors
- with nobody

When it comes to the topic of energy and energy saving, how strongly do you trust the information of the following individuals and institutions?
On the scale from 1 (no confidence) to 7 (very high confidence), indicate how strong your confidence is.

	1 (no confidence)	2	3	4	5	6	7 (very high confidence)
Friends and acquaintances							
Family							
Federal Office of Energy							
Stadtwerk Winterthur							
Energiefachstelle der Stadt Winterthur							
Science							
Environmental organizations							
Work and study colleagues							
House administration							
Caretaker							

32 Questions on the current situation

At the end of the survey you will be asked a few questions about your person and your current life situation.

Do you posess the property or apartment in which you live?

- yes
- no

How many people live in your household (you included)?

1
2
3
4
5
6
7
8
9
> 9

Do minor children in your household?

- yes
- no

What is the size of your apartment / house (without screed, cellar, balcony)?

-- don't know--<
31 m2
31-40 m2
41-50 m2
51-60 m2
61-70 m2
71-80 m2
81-90 m2
91-100 m2
101-110 m2
111-120 m2
121-130 m2
131-140 m2
141-150 m2
151-160 m2
161-170 m2
171-180 m2
> 180 m2

What kind of electricity product do you buy at home?

- e-Strom.Gold
- e-Strom.Silber
- e-Strom.Bronze
- e-Strom.Weiss
- e-Strom.Grau
- don't know
- other

Which means of transport do you use most frequently (by number of ways)?

Bicycle
Electric bicycle
Moped
Scooter
Motorcycle
Automobile
Public transport
- other -

How far is the distance from your home to your place of work (or place of study, etc.)?

If you do not know the distance exactly, we ask you to estimate it.

- Number of kilometers (please enter only numbers):
- do not commute

Do you or a person in your household have a car?

- yes
- no

Do you have a subscription (without a Halbtax) for public transport (e.g., GA, ZVV subscription, etc.)?

- yes
- no

How many times did you fly for private purposes in 2014 (back and forth is considered twice)?

never
1 time
2 times
3 times
4 times
5 times
6 times
more than 6 times

How many days a week do you usually eat meat?

never
< 1
1
2
3
4
5
6
7

How often do you buy / receive a new mobile phone?

- I do not have a mobile phone -
More than once a year
Once a year
every second year
Every third year
Every fourth year
Less than every fourth year
My current mobile phone is my only one

How environmentally conscious do you value yourself?

not at all ○ ○ ○ ○ ○ ○ ○ very much

33 Demographic questions

In which year were you born?

1917
1918
1919
1920
1921
1922
1923
1924
1925
1926
1927
1928
1929
1930
1931
1932
1933
1934
1935
1936
1937
1938
1939
1940
1941
1942
1943
1944
1945
1946
1947
1948
1949
1950
1951
1952
1953
1954
1955
1956
1957
1958
1959
1960
1961
1962
1963
1964
1965
1966
1967
1968
1969
1970
1971
1972
1973

What is your highest level of education?

No school graduation
Compulsory school
An apprenticeship, household apprenticeship
Apprenticeship
Vocational school, matura school
Master's examination, technical college and
University of applied sciences, university, ETH
other education

What is your profession?

What is the monthly net income (income after deductions) of your household (sum of all your household income)

-- not specified –
< 4'000 CHF.
4'001 - 6000 CHF.
6'001 - 8000 CHF.
8'001 - 10000 CHF.
10'001 - 12'000 CHF.
12'001 - 14'000 CHF.
14'001 - 16'000 CHF.
16'001 - 18'000 CHF.
> 18'000 CHF.

How do you assess your political attitude?

left ○ ○ ○ ○ ○ ○ ○ right

Which political party represents your interests most closely?

-- not specified --
-- no party --
AL - Alternative Liste
CVP - Christlichdemokratische Vo
FDP - Freisinnig-Demokratische P
Grüne / Junge Grüne
SVP - Schweizerische Volkspartei
SP - Sozialdemokratische Partei
Piraten - Piratenpartei
GLP - Grünliberale Partei
EVP - Evangelische Volkspartei
EDU - Eidgenössisch-Demokratis
other

Are you an active member of one or more club(s)?

Please tick all that apply:

- ☐ No
- ☐ Yes, in the field of sports
- ☐ Yes, in the field of music
- ☐ Yes, in the field of politics
- ☐ Yes, in the field of environment
- ☐ Yes, in another area

34 Comments and suggestions

Thank you for your participation

Please click Next to receive your intervista bonus points.

35 Final page
